# Monthly Alternating Injections of Aflibercept and Bevacizumab for Neovascular Age-Related Macular Degeneration

**DOI:** 10.3390/jcm11061543

**Published:** 2022-03-11

**Authors:** Junyeop Lee, You Na Kim, June-Gone Kim

**Affiliations:** Department of Ophthalmology, Asan Medical Center, University of Ulsan College of Medicine, Seoul 05505, Korea; j.lee@amc.seoul.kr (J.L.); youna.kim.oph@gmail.com (Y.N.K.)

**Keywords:** aflibercept, age-related macular degeneration, alternating injection, bevacizumab

## Abstract

We investigated the efficacy of monthly alternating injections of aflibercept and bevacizumab (MAAB) for maintenance treatment in patients with neovascular age-related macular degeneration (AMD) who showed improvement with the initial monthly injections but presented with rapid worsening after conversion to bimonthly injections. We included 72 patients with neovascular AMD who showed improvement with loading injections of aflibercept. For maintenance treatment, bevacizumab was administered every alternate month between the bimonthly aflibercept injections in 24 (33.3%) eyes showing worsening (MAAB group). The other eyes were treated with aflibercept (BiA group) bimonthly. Baseline low retinal thickness, thick choroid, and presence of intraretinal fluid were associated with worsening after extending the injection intervals. Visual improvement was lower in the MAAB group than in the BiA group, but the final visual outcomes were comparable. Additional bevacizumab stabilized the early fluctuation of retinal thickness, thus maintaining long-term visual stability without increasing the risk of geographic atrophy or disciform scar until the second year. Previously treated eyes or those with polypoidal choroidal vasculopathy responded less to the initial loading doses and showed worsening under the bimonthly regimen. MAAB was effective in preventing anatomical and functional deterioration when bimonthly aflibercept proved insufficient for the maintenance treatment of neovascular AMD.

## 1. Introduction

Age-related macular degeneration (AMD) is a leading cause of blindness worldwide. Neovascular AMD is a condition in which abnormal blood vessels grow under the retina and leak fluid. It is an advanced form of AMD where the exudation and scar formation under the chronic inflammation damage the photoreceptor, giving rise to rapid and severe vision loss. [1]. Vascular endothelial growth factor (VEGF) is a major factor that stimulates vision-threatening neovascular AMD [1]. Several anti-VEGF agents that prevent severe vision loss in patients with neovascular AMD are widely available [2]. Aflibercept is a recombinant fusion protein consisting of the extracellular domains of human VEGF receptors with substantially higher affinity than conventional soluble VEGF receptors [3]. In contrast, bevacizumab is a humanized anti-VEGF monoclonal antibody [2]. Compared to either ranibizumab or bevacizumab, aflibercept has better pharmacokinetic profile and greater binding affinity to VEGF-A. Thus, it has been approved as the current regimen and consists of three initial 2-mg monthly injections followed by a bimonthly dosing regimen [4]. Pivotal trials demonstrated that bimonthly aflibercept was not inferior to monthly ranibizumab in maintaining vision in treatment-naïve neovascular AMD [4,5]. However, several studies revealed that a majority of the eyes showed anatomical and functional worsening after the extension of the treatment interval to 2 months in clinical practice [6,7,8,9]. These reports suggested that bimonthly aflibercept dosing may not be optimal for maintenance in some populations of patients, even though they responded well to the initial loading regimen. There is no possibility of continuing monthly aflibercept over three months under the registration regulations in several countries or is not covered by health insurance reimbursements. Thus, it would be desirable to modify the regimen or combine aflibercept with other agents in cases in which the bimonthly aflibercept regimen could not achieve stable maintenance. The present study was designed to identify subpopulations that experienced anatomical and functional worsening, thereby requiring additional injections when aflibercept dosing interval was increased to 2 months, as well as to investigate the efficacy and safety of monthly alternating injections of aflibercept and bevacizumab for the maintenance treatment of neovascular AMD.

## 2. Materials and Methods 

### 2.1. Study Design 

This retrospective, consecutive case series was approved by the Institutional Review Board of Asan Medical Center and Yeungnam University Hospital. The study adhered to the tenets of the Declaration of Helsinki. Off-label use of intravitreal bevacizumab was approved by Institutional Review Board of both institutes. Informed consent for each injection was obtained from the subjects.

### 2.2. Patients 

The present study included patients with neovascular AMD presenting between March 2016 and March 2017, at Asan Medical Center and Yeungnam University Medical Center. The study included patients older than 50 years who received three initial loading injections of aflibercept and showed improvement with the initial loading doses, which were part of the maintenance regimen eligible for reimbursement under the Korean National Health Insurance system. Patients with a history of vitrectomy, laser photocoagulation, or photodynamic therapy (PDT), as well as those with other retinal diseases such as angioid streaks, vitelliform macular dystrophy, and retinal vein or artery occlusion were excluded.

### 2.3. Interventional Procedures 

All patients received three monthly loading doses of aflibercept (Eylea, Regeneron Pharmaceuticals, Inc., Terrytown, NY, USA) (2 mg, 0.05 mL) injected intravitreally via the pars plana under sterile conditions. After three monthly injections, the patients were divided into two groups according to the change in the retinal fluid status or hemorrhage from month 3 to 4: the bimonthly aflibercept (BiA) group or the monthly alternating injections of aflibercept and bevacizumab (Avastin, Genentech, South San Francisco, CA, USA) (1.25 mg, 0.05 mL) (MAAB) group. If the previous subretinal or intraretinal fluid increased or new fluid or hemorrhage occurred from month 3 to 4, the patients were categorized into the MAAB group, and they were treated with additional bevacizumab injections between the bimonthly aflibercept injections as a maintenance treatment that was part of a fixed regimen. Otherwise, the patients were treated with bimonthly aflibercept injections only (BiA). Follow-up was performed every month in the MAAB group, and every 2 months in the BiA group until 12 months. 

After 12 months, the treat-and-extend (TAE) aflibercept injection protocol in both groups was adopted according to the recommendations of the Korean National Health Insurance system. If the exudative changes, including the presence of intraretinal and/or subretinal fluid and/or hemorrhage, were absent for more than 2 months, the aflibercept injection interval was extended by 2 weeks to up to 3 months. 

**Figure 1:**
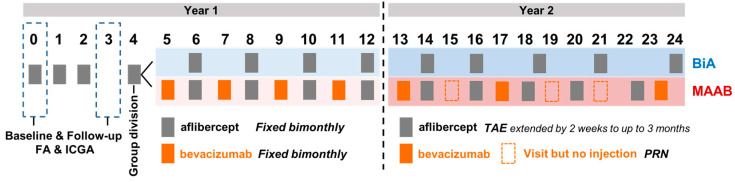
Treatment protocols. BiA = bimonthly aflibercept, MAAB = monthly alternating injections of aflibercept and bevacizumab, TAE = treat-and-extend, PRN = pro re nata.

In the second year of treatment in the MAAB group, additional bevacizumab injections were employed only if the level of retinal fluid or hemorrhage was higher than that at the previous visit as a pro re nata (PRN) regimen at every monthly visit. The treatment protocols are summarized in Figure 1.

### 2.4. Ophthalmological Examinations

All patients underwent best-corrected visual acuity (BCVA) measurement with the refraction test throughout the course of treatment. BCVA was converted to the logarithm of the minimum angle of resolution (LogMAR) units. Fluorescein angiography and ICG angiography were performed using a Heidelberg retinal angiography system (Heidelberg Engineering, Heidelberg, Germany) to diagnose AMD and determine its subtypes, including typical AMD characterized by the choroidal neovascular membrane, polypoidal choroidal vasculopathy (PCV), and retinal angiomatous proliferation. We acquired optical coherence tomography (OCT) images by using the Heidelberg Spectralis OCT system (Heidelberg Engineering, Germany) at every visit.

### 2.5. Main Outcome Measures

The primary outcome was changes in BCVA in the MAAB and BiA groups during 24 months. The mean changes in central retinal thickness (CRT) from baseline up to 24 months were compared between the two groups. The baseline factors potentially influencing the likelihood of worsening were analyzed after the aflibercept injection interval was increased to 2 months. The secondary outcome was the retinal fluid status during the maintenance period in both the groups. Subgroup analysis in terms of visual and anatomical changes was performed according to the subtypes of neovascular AMD and previous treatment history.

### 2.6. Statistical Analysis

All statistical analyses were performed using IBM SPSS Statistics for Windows/Macintosh, Version 24.0 (IBM Corp., Armonk, NY, USA). Categorical variables were presented as numbers and percentages and were analyzed using the chi-square test or Fisher’s exact test. The Wilcoxon signed-rank test was used to compare the mean BCVA or CRT from baseline. Continuous variables, presented as the mean, standard deviation, and range, were analyzed using the two-tailed unpaired *t*-test or a generalized linear model to determine the relationships between the parameters and outcomes. A *p*-value < 0.05 was considered statistically significant.

## 3. Results

### 3.1. Patient Demographics and Baseline Characteristics

Seventy-two eyes of 72 patients with neovascular AMD that showed improvement during the initial aflibercept loading injections were included and followed up for 24 months. Among the 72 eyes, 24 (33.3%) showed worsening of any fluid after increase of the treatment interval to 2 months, and these eyes were categorized into the MAAB group. The remaining 48 eyes (66.7%) were treated with bimonthly aflibercept injections (BiA group). The baseline characteristics of the MAAB and BiA groups are summarized in Table 1. Compared to the BiA group, the MAAB group had younger patients with a male predominance. Patients previously treated using anti-VEGF agents, eyes with PCV, and eyes with intraretinal fluid (IRF) were more frequent in the MAAB group than in the BiA group. The MAAB group had better BCVA, lower retinal thickness, and higher subfoveal choroidal thickness at baseline, as described in Table 1.

### 3.2. Comparison between the Two Groups after the Three Monthly Loading Injections of Aflibercept

As the present study enrolled patients who showed improvement with the three loading injections of intravitreal aflibercept, the mean BCVA and CNV size improved in the third month from baseline in both the MAAB and BiA groups, with no significant intergroup differences, as described in Table 2. Compared to the BiA group, the MAAB group showed less improvement in CRT and pigment epithelial detachment (PED) during the loading regimen. One month after the three loading injections, all 48 eyes in the BiA group acquired a dry macula status, but one-third of the eyes (8 of 24) in the MAAB group displayed incomplete fluid absorption. These data suggest the lower treatment efficacy of aflibercept during the loading regimen in the MAAB group than in the BiA group.

### 3.3. Visual Outcomes from Baseline to the 24th Month 

**Figure 2:**
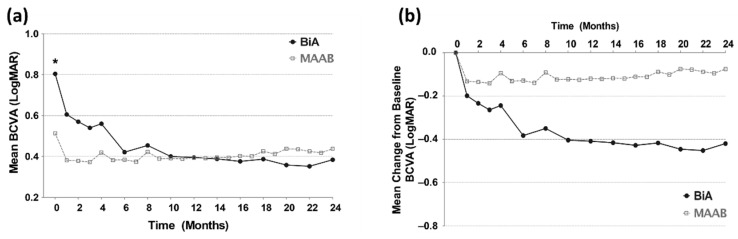
Visual outcome from baseline to the 24th month. (**a**) Compared to the BiA group, the MAAB group has better BCVA at baseline. * *p* = 0.001 by two-tailed unpaired *t*-test. (**b**) The BiA group shows greater improvement in BCVA than does the MAAB group.

In both the groups, the mean BCVA at every follow-up visit improved from that at baseline and remained stable for 2 years (Figure 2). Compared to the BiA group, the MAAB group had better baseline BCVA (*p* = 0.001, two-tailed unpaired *t*-test), but no significant difference was observed in BCVA at the follow-up visits between the two groups. When the injection interval was increased to 2 months, visual acuity in the fourth month was worse than that in the third month in both the groups. Although the BiA group showed more improvement in BCVA for 2 years than did the MAAB group (−0.420 vs. −0.075 LogMAR), both the groups maintained visual stability without any fluctuation or deterioration of visual function. Visual outcomes in the first and second year were comparable between the two groups. Even in the second year, when the fixed regimens were changed into the TAE aflibercept and PRN bevacizumab regimens, stable visual acuity was maintained until the second year (Figure 2).

### 3.4. Anatomical Outcomes from Baseline to the 24th Month 

**Figure 3:**
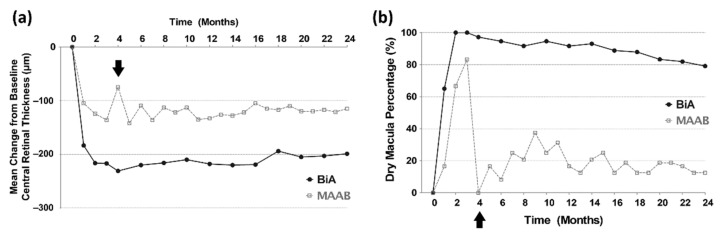
Anatomical outcomes from baseline to the 24th month. (**a**) MAAB group shows rapid worsening at the fourth month. (**b**) The BiA group maintains a higher percentage of dry macula than does the MAAB group over the entire period. Arrows indicate rapid anatomical worsening at the fourth month in the MAAB group.

The mean CRT at every follow-up visit decreased from that at baseline and remained stable for 2 years in both the groups (Figure 3). Compared to the BiA group, the MAAB group showed less improvement in CRT during the loading phase and showed worsening in the fourth month. These data suggest the lower efficacy and shorter durability of aflibercept in the MAAB group. The BiA group maintained a higher percentage of dry macula than did the MAAB group during the entire period. Fluctuations in CRT were observed in the MAAB group in the first year, suggesting the lower efficacy of bevacizumab to that of aflibercept. Additional bevacizumab injections could not fully restore the anatomical worsening that occurred when the injection interval was increased to 2 months after the three loading doses in the first year. However, this fluctuation stabilized in the second year (Figure 3). Development of geographic atrophy (3 vs. 5, *p* = 0.538) and disciform scar (6 vs. 14, *p* = 0.710) were not different in the MAAB and BiA groups. There was no serious adverse event including endophthalmitis or systemic vascular diseases in both groups.

### 3.5. Subgroup Analyses

**Figure 4:**
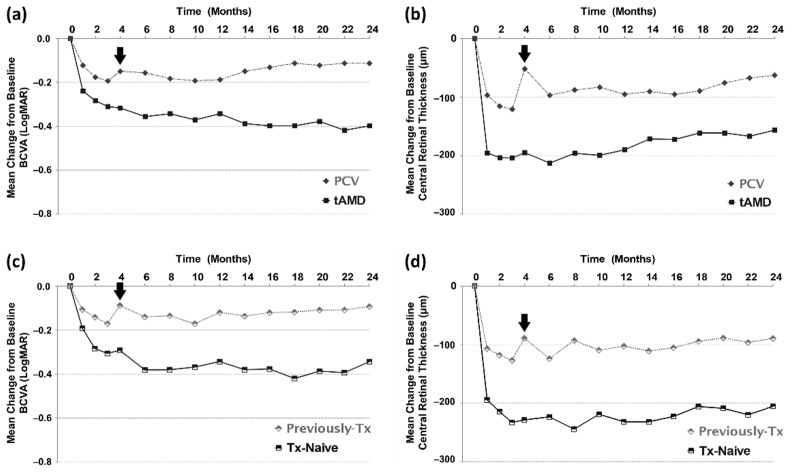
Subgroup analyses according to the subtypes of AMD and previous treatments. The eyes with PCV (**a**,**b**) and previously treated eyes (**c**,**d**) show less response to the three initial loading doses, and show much worsening when the injection interval is increased at the fourth month (arrows).

In the subgroup comparisons, compared to eyes with typical AMD or treatment-naïve eyes, eyes with PCV and previously treated eyes presented with lower response to the three initial loading doses and showed much worsening when the injection interval was increased to 2 months (in the fourth month) in terms of BCVA and CRT (Figure 4). In addition, visual and anatomical improvements were lower in eyes with PCV and previously treated eyes than in eyes with typical AMD or treatment-naïve eyes. These data indicated that eyes with PCV and previously treated eyes had a possibility of rapid worsening after the extension of the injection interval, even though they responded well to the loading injections.

### 3.6. Change in Treatment Protocols in the Second Year

**Figure 5:**
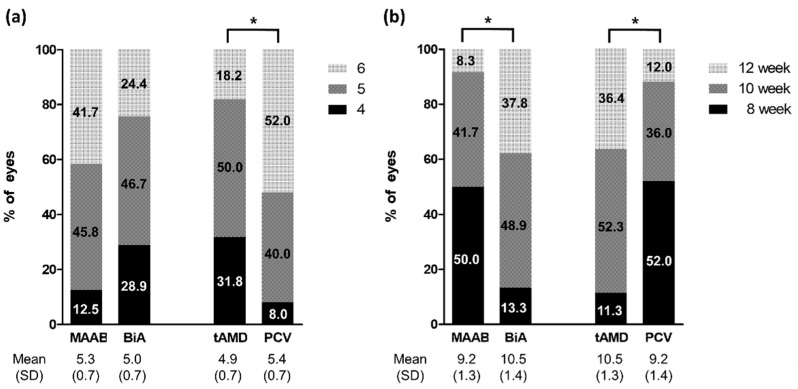
Number of aflibercept injections and last treatment interval in the second year. (**a**) Number of aflibercept injections during the second-year TAE/PRN period is not significantly different between the groups, but more injections are required in eyes with PCV than in those with typical AMD. (**b**) The treatment intervals are significantly extended in the BiA or typical AMD groups than in the MAAB or PCV groups. * *p* < 0.05 by chi-square test.

In the second year, the treatment regimens were changed from the fixed bimonthly protocol to the TAE aflibercept and PRN bevacizumab regimens to reflect real-world treatment circumstances. The present study investigated whether the injection interval could be increased in the MAAB and BiA groups. In the first year, eight aflibercept injections including the loading injections were performed in all patients, and four bevacizumab injections were additionally performed in the MAAB group. In the 2nd year, the number of aflibercept injections was the same in the MAAB and BiA groups (5.3 vs. 5.0, *p* = 0.188, Figure 5a). However, the last aflibercept injection interval in the 24th month was shorter in the MAAB group than in the BiA group (9.2 vs. 10.5 week, *p* = 0.001, Figure 5b). In the MAAB group, eyes with typical AMD needed fewer aflibercept and bevacizumab injections than did eyes with PCV in the second year, as described in Table 3. In contrast, in the BiA group, no significant difference was observed in the number of aflibercept injections between eyes with typical AMD and PCV, as shown in Table 3.

## 4. Discussion

The results of the present study indicate that one-third of the patients with neovascular AMD who responded well to the loading injections of intravitreal aflibercept showed rapid worsening after the extension of the injection interval to 2 months. The findings of the present study are comparable to that of previous studies that reported that 28–31% of eyes showed anatomical worsening after the extension of the treatment interval [6,9]. The worsening that occurred when the injection interval was increased did not fully recover and resulted in an unstable retinal fluid status during the first 12 months. The results of the present study were consistent with the oscillating or saw-tooth pattern of retinal thickness seen in the VIEW studies [4]. The VIEW trials revealed that this CRT fluctuation pattern remained until 96 weeks, and the improved visual gain during the first year continued to diminish in the second year [5]. Therefore, these data suggest that the regimen has to be modified for achieving stable maintenance. 

To the best of our knowledge, this is the first real world study demonstrating that the MAAB regimen could be effective in preventing anatomical and functional deterioration without increasing the risk of geographic atrophy when bimonthly aflibercept is insufficient for the maintenance treatment. In several countries, aflibercept is not used every month in the poorly responding subgroup under the drug’s governmental guidelines or registration regulations or is not covered by health insurance reimbursements. Our study indicated that the MAAB regimen can stabilize the early fluctuation in retinal thickness. The visual gain achieved during the loading phase was maintained for up to 2 years, even though the fixed regimens were changed into proactive and as-needed regimens in the second year. The as-needed or TAE regimens, rather than continuous monthly or bimonthly injections, tend to be used in daily clinical practice [10]. The second-year data in the present study demonstrated that the modified MAAB regimen comprising proactive aflibercept and PRN bevacizumab injections was also effective as a maintenance treatment in the real-world practice.

The underlying mechanism associated with the lower efficacy and shorter durability of 2 months of aflibercept remains unclear. Aflibercept is a soluble decoy receptor with substantially higher affinity than conventional soluble VEGF receptors [11]. Aflibercept has a markedly higher affinity for VEGF-A [3]. However, there is still controversy surrounding its affinity [12,13]. Moreover, the vitreous half-life of aflibercept is shorter than that of bevacizumab [14]. These evidence account for the possibility of the limited efficacy of aflibercept in subpopulations with neovascular AMD. In the real-world, bevacizumab, ranibizumab, and aflibercept achieved a comparable visual outcome for treating neovascular AMD [15].

As aflibercept and bevacizumab differ in their molecular structure and properties, both agents could function complementarily to suppress exudation in neovascular AMD. Multiple reports have demonstrated that patients with bevacizumab tachyphylaxis respond positively to aflibercept [16]. In contrast, switching from aflibercept to bevacizumab treatment has an equivalent anatomical effect in eyes with neovascular AMD as does switching from bevacizumab to aflibercept [17,18]. Concerns exist about the potential over-dosing issue when using multiple anti-VEGF agents. However, biweekly bevacizumab injections are effective for the treatment of non-responding neovascular AMD with no reported adverse events [19]. Thus, treatment efficacy could vary in patients with AMD, and personalized treatments are required. 

Notably, if anatomical worsening occurred in the fourth month, it was not fully restored despite a switch to the monthly alternating injection schedule and was associated with poor visual improvement in the present study. Thus, identifying the risk groups and maintaining the monthly dosing interval are necessary to maintaining the dry macula which leads to long-term visual improvement. The present study demonstrated that low retinal thickness, high choroidal thickness, and the presence of intraretinal fluid at baseline were associated with rapid worsening after the extension of aflibercept injection intervals. In addition, eyes with PCV and previously treated eyes are more resistant to the aflibercept loading regimen and tend to showing worsening when the injection interval is extended. In these cases, we should refrain from extending the aflibercept treatment interval or from adding other treatment agents from the third month.

Factors associated with a poor response to or shorter durability of aflibercept in neovascular AMD remain unclear. Higher body mass index and geographic atrophy are considered potential indicators for a poor response to aflibercept [20]. Remnant polyps and PED morphology are associated with recurrence of PCV after aflibercept treatment [21]. Importantly, serous PED and fibrovascular PED were associated with non-responsiveness to treatment [22]. In the present study, the maximum height of PED showed worsening when the aflibercept injection interval was increased. One previous study reported that a greater decrease in subfoveal choroidal thickness is associated with a good response to aflibercept in both eyes with typical AMD and PCV [23]. The findings of the present study are consistent with those of this previous study in that the MAAB group had higher choroidal thickness at baseline and showed less decrease than did the BiA group after the loading phase. The presence of IRF at baseline was related to worsening in the present study. In a previous study, eyes with inner nuclear layer cystoid spaces also showed worse visual outcomes than did those without [24]. The presence of IRF might be a degenerative cystic change rather than an exudative change.

PCV is considered to respond differently to intravitreal anti-VEGF treatments than does typical AMD [25,26]. One prospective case series including patients with treatment-naïve PCV demonstrated retinal fluid recurrence in approximately one-third of the eyes after the treatment interval was extended to 2 months [27]. Therefore, the conventional bimonthly maintenance dosing schedule is suboptimal for some patients, and more intensive treatments are warranted. The present study also clarified that eyes with PCV or previously treated eyes presented much worsening when the injection interval was increased. In the second year, i.e., the extension period, eyes with PCV required more frequent injections for maintenance, especially in cases presenting rapid worsening after conversion to the bimonthly regimen.

The present study had several limitations. All patients were Korean. Selection bias can occur in such a retrospectively designed study. Therefore, there was several differences in baseline characteristics between groups. It was not ethically appropriate to divide the worsening group into two independent groups with or without additional bevacizumab. This study lacks the proper control in each group according to the progression in the second year. Moreover, the present study did not control for the types and numbers of anti-VEGF treatments before the eyes were enrolled in the study. As only one treatment regimen was used, the potential value of other regimens, such as those combined with ranibizumab and/or PDT, was not examined. Despite these limitations, the present study included well-designed treatment protocols and extensive follow-up examinations similar to a prospective randomized trial.

## 5. Conclusions

The present study revealed that, for the first time, the monthly alternating injections of aflibercept and bevacizumab stabilized the early fluctuation of retinal thickness, and thus maintained long-term visual stability in patients with neovascular AMD who presented with rapid worsening after the treatment interval was increased to 2 months. Therefore, under the circumstance that monthly aflibercept is not available, the MAAB regimen could be considered for preventing anatomical and functional deterioration when bimonthly aflibercept injections are not sufficient for the maintenance treatment of neovascular AMD.

## Figures and Tables

**Table 1:** Demographics and baseline characteristics.

	Total	MAAB Group	BiA Group	*p* Value
Age (years)	68.8 ± 10.1	65.1 ± 6.8	70.6 ± 10.9	0.012
Sex (M/F)	42/30	18/6	24/24	0.048
Lens (phakic/pseudophakic)	56/16	22/2	34/14	0.070
Previous treatments (previously treated/treatment-naïve)	32/40	18/6	14/34	0.001
Refractive error (diopter)	0.090 ± 1.280	0.000 ± 1.552	0.135 ± 1.137	0.675
Baseline BCVA (Log MAR)	0.707 ± 0.394	0.513 ± 0.209	0.804 ± 0.429	0.001
**Baseline Angiographic finding**				
Type of AMD (tAMD/PCV/RAP)	44/25/3	9/15/0	35/10/3	0.002
Area of CNV (mm^2^)	0.904 ± 1.066	1.426 ± 0.689	0.631 ± 1.131	0.001
**Baseline OCT findings**				
CRT (um)	437.5 ± 121.4	386.7 ± 68.9	462.9 ± 134.1	0.002
Maximum height of PED (um)	238.1 ± 143.9	246.7 ± 120.7	233.8 ± 155.24	0.724
Subfoveal choroidal thickness (um)	239.7 ± 86.3	282.8 ± 95.1	218.1 ± 73.55	0.002
Presence of IRF (yes/no)	44/28	23/1	21/27	0.001
Presence of SRF (yes/no)	72/0	24/0	48/0	NA

BiA = bimonthly aflibercept, MAAB = monthly alternating injections of aflibercept and bevacizumab, BCVA = best-corrected visual acuity, CNV = choroidal neovascularization, PCV = polypoidal choroidal vasculopathy, tAMD = typical age-related macular degeneration, RAP = retinal angiomatous proliferation, CRT = central retinal thickness, PED = pigment epithelial detachment, IRF = intraretinal fluid, SRF = subretinal fluid.

**Table 2:** Changes after three monthly loading injections of aflibercept.

	Total	MAAB Group	BiA Group	*p* Value
Δ BCVA	−0.038 ± 0.239	−0.042 ± 0.156	−0.035 ± 0.273	0.918
**Angiographic findings**				
CNV area (3 month/baseline, fold)	0.49 ± 0.22	0.54 ± 0.25	0.48 ± 0.21	0.336
**OCT findings**				
Δ CRT (um)	−185.1 ± 136.0	−124.4 ± 72.1	−216.4 ± 150.1	0.001
PED height (3 month/baseline, fold)	0.63 ± 0.41	0.87 ± 0.17	0.51 ± 0.44	0.001
Choroidal thickness (3 month/baseline, fold)	0.95 ± 0.16	0.91 ± 0.16	0.98 ± 0.17	0.072
Dry macula (N, %) at 3 month	64 (88.9)	16 (66.7)	48 (100)	0.001

BiA = bimonthly aflibercept, MAAB = monthly alternating injections of aflibercept and bevacizumab, BCVA = best-corrected visual acuity, CNV = choroidal neovascularization, CRT = central retinal thickness, PED = pigment epithelial detachment.

**Table 3:** Number and interval of injections in the second year.

	MAAB Group			BiA Group		
	tAMD (N = 9)	PCV (N = 15)	*p* Value	tAMD (N = 35)	PCV (N = 10)	*p* Value
Number of aflibercept injection in Year 2	4.7 ± 0.5	5.7 ± 0.5	0.001	4.9 ± 0.7	5.1 ± 0.7	0.489
Last aflibercept injection interval (wk)	10.0 ± 1.4	8.7 ± 1.0	0.012	10.6 ± 1.2	10.0 ± 1.5	0.200
Number of bevacizumab injection Year 2	2.9 ± 0.8	5.6 ± 0.5	0.001	NA	NA	NA

BiA = bimonthly aflibercept, MAAB = monthly alternating injections of aflibercept and bevacizumab, tAMD = typical age-related macular degeneration, PCV = polypoidal choroidal vasculopathy.

## Data Availability

The data presented in this study are available on request from the corresponding author.
